# Heparin-Induced Thrombocytopenia Causing Early Venous Thrombosis and Free Flap Failure After Mandibular Reconstruction: A Case Report

**DOI:** 10.7759/cureus.98630

**Published:** 2025-12-07

**Authors:** Khalid Abdel Aziz, Faris Abdon, Mohamed Salim

**Affiliations:** 1 Plastic and Reconstructive Surgery Department, Al Qassimi Hospital, Sharjah, ARE; 2 Medical Sciences Department/Medical Biochemistry Unit, Orotta College of Medicine and Health Sciences, Asmara, ERI; 3 Plastic Surgery Department, Sharg Alneel Teaching Hospital, Khartoum, SDN

**Keywords:** 4ts score, argatroban, direct thrombin inhibitor, free flap, free tissue transfer, heparin-induced thrombocytopenia, mandibular reconstruction, microvascular thrombosis, platelet factor 4 antibodies

## Abstract

Heparin-induced thrombocytopenia (HIT) is an under-recognized cause of early microvascular thrombosis and free flap loss after head and neck reconstruction. We report a 45-year-old man who underwent fibula free flap mandibular reconstruction and, within 72 hours of perioperative heparin exposure, developed recurrent venous thrombosis and persistent flap congestion despite multiple re-explorations. The platelet count fell markedly, the 4Ts score indicated a high pretest probability of HIT, and anti-platelet factor 4/heparin testing subsequently returned a positive result. Despite critical care escalation, the patient progressed to multiorgan failure and died on postoperative day 7. This case underscores that in microsurgical patients, repeated venous thrombosis with preserved inflow should prompt the immediate consideration of HIT: stop all heparin (including flushes), begin a non-heparin anticoagulant while arranging confirmatory testing, and involve hematology early. We outline a practical timeline, platelet fall, 4Ts scoring, and differential diagnosis for postoperative thrombocytopenia to aid in the rapid recognition and management of similar cases.

## Introduction

Microvascular tissue transfer remains the gold standard in oral cavity reconstruction [1,2]. It has a high success rate (95-99%) [3], yet unpredictable complications such as heparin-induced thrombocytopenia (HIT) can severely compromise outcomes [4]. HIT is an immune-mediated disorder characterized by antibodies that target complexes of heparin and platelet factor 4 (PF4) [5,6], causing profound platelet activation and a hypercoagulable state [7]. Although rare, with an incidence ranging from 0.2% to 5% in patients receiving heparin, HIT remains significantly underdiagnosed and may result in catastrophic thrombotic events [8].

Mandibular free flap reconstruction is increasingly planned with multi-step virtual workflows (e.g., virtual surgical planning with cutting guides and staged osteotomies), which can lengthen operations and complicate postoperative surveillance. In such pathways, systemic causes of thrombosis, like HIT, may be overlooked if flap congestion is attributed solely to anastomotic or mechanical factors. Contemporary reviews of virtual surgical planning in maxillofacial reconstruction highlight the complexity of the workflow and the need for structured perioperative monitoring [9,10].

A unique aspect of HIT is the timing of the immune response, which typically occurs within 5-14 days post-heparin exposure but can manifest rapidly upon re-exposure to heparin within hours [8]. Early clinical recognition (guided by the widely recommended 4Ts scoring system (thrombocytopenia, timing of platelet count fall, thrombosis, and exclusion of other causes) by Warkentin and Heddle [11]) is crucial to improve outcomes through prompt diagnosis and initiation of alternative anticoagulation therapies [4].

## Case presentation

A 45-year-old male patient, a heavy smoker without significant prior medical history, presented to our clinic at Sharg Alneel Teaching Hospital, Khartoum, Sudan, for the reconstruction of mandibular and soft tissue defects resulting from a gunshot injury two years earlier (Appendix A). Pre- and postoperative hematology/coagulation values are summarized in Table 1; preoperative electrocardiogram (ECG) and chest radiography were unremarkable (Appendix B).

**Table 1:** Preoperative and postoperative day 5 hematology and coagulation values with institutional reference intervals Abnormal values are emphasized. Reference intervals reflect the testing laboratory's ranges at the time of analysis. CBC: complete blood count; WBC: white blood cells; RBC: red blood cells; MCV: mean corpuscular volume; MCH: mean corpuscular hemoglobin; MCHC: mean corpuscular hemoglobin concentration; PT: prothrombin time; aPTT: activated partial thromboplastin time; INR: international normalized ratio

Test	Preoperative CBC value	Postoperative day 5 value	Reference interval
WBC	6.2×10^9^/L	7.5×10^9^/L	2.5-8.0×10^9^/L
RBC	5.2×10^12^/L	4.6×10^12^/L	4.6-6.3×10^12^/L
Hemoglobin	15.1 g/dL	13.8 g/dL	12.7-18.6 g/dL
Hematocrit	45.8%	41%	41.8-57.5%
MCV	88 fL	89 fL	81.6-101 fL
MCH	29 pg	30 pg	24.4-33 pg
MCHC	32.5 g/dL	33 g/dL	29.6-33 g/dL
Platelets	240×10^9^/L	42×10^9^/L	85.4-379×10^9^/L
Neutrophils %	57%	58%	26.9-62%
Lymphocytes %	32%	30%	26.8-61.8%
Monocytes %	8.1%	8%	2-10%
Eosinophils %	1.9%	2%	1.12-3.4%
Basophils	1%	2%	0.5-1%
PT	12.2 s	13.8 s	10-13.4 s
aPTT	29.5 s	48.9 s	20.4-35.3 s
INR	1.0	1.9	0.8-1.2 ratio

**Figure 1:**
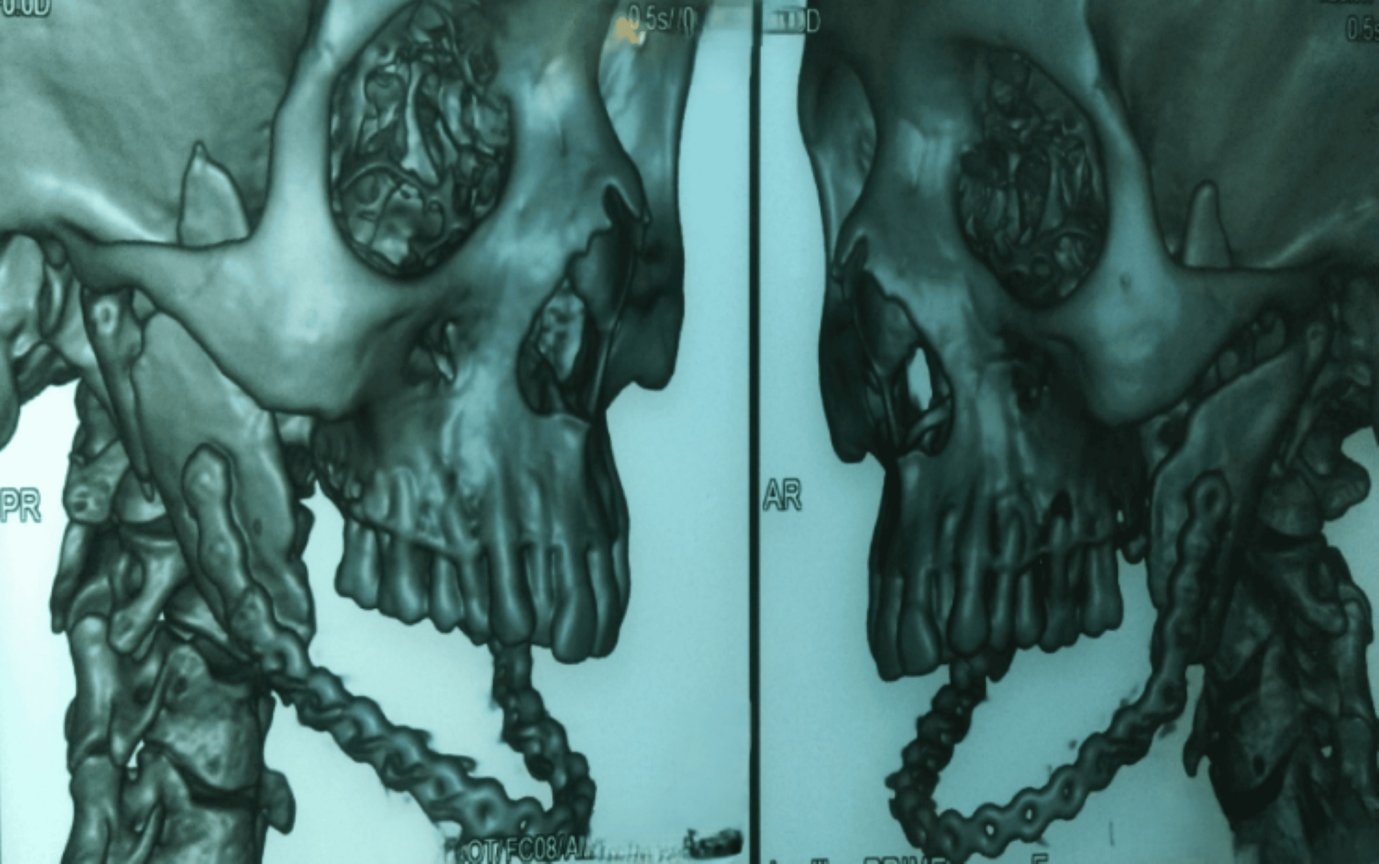
Preoperative 3D CT showing a segmental mandibular defect and plate Volume-rendered CT demonstrating the mandibular continuity defect spanned by a temporary reconstruction plate placed for interim stabilization before fibular osteocutaneous free flap reconstruction.

Preoperative volume-rendered 3D CT demonstrated the segmental mandibular continuity defect spanned by a temporary reconstruction plate (Figure 1).

**Figure 2:**
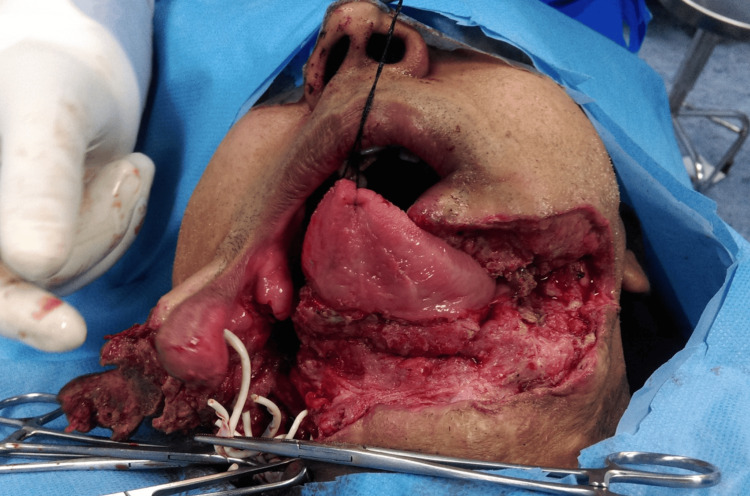
Initial intraoperative exposure and removal of the temporary mandibular plate At the start of the operation, after cervical exposure of the mandibular defect, the previously placed load-bearing plate and screws were removed, and fibrous tissue was debrided to prepare the recipient bed for flap inset. This image represents initial exposure and site preparation, not a re-exploration.

The reconstruction procedure involved harvesting a free fibular osteocutaneous flap from the left leg (Appendix C). At the start of the operation, following cervical exposure, the temporary load-bearing mandibular plate and screws were removed, and the recipient bed was prepared for flap inset (Figure 2).

An intravenous bolus of heparin (5000 IU) was administered preoperatively, and intraoperative vessel preparation included irrigation with heparinized saline. The arterial inflow and venous outflow of the flap were established through microsurgical anastomoses to the superior thyroid artery and veins (superior thyroid and anterior jugular veins). The surgery proceeded uneventfully, and the patient received two units of packed red blood cells intraoperatively.

Postoperative management involved standard care, with 5000 IU of subcutaneous heparin administered twice daily and hourly clinical and Doppler ultrasound monitoring of the flap. Venous congestion first appeared on postoperative day 3 and persisted despite re-exploration. Initial conservative management involved repeated needle punctures to facilitate venous drainage, yet the congestion persisted, characterized by bluish discoloration, reduced temperature, and dark-colored blood on puncture. Despite persistent Doppler signals indicating adequate arterial perfusion, the flap required surgical re-exploration. Multiple thrombi were identified in the venous anastomoses during re-exploration, necessitating mechanical thrombectomy, after which venous drainage partially improved. Operative timings, venous anastomotic findings (including multiple intraluminal thrombi), and actions taken are detailed in Appendix D. Despite these interventions, congestion recurred, prompting three additional explorations over the subsequent 48 hours, each demonstrating adequate arterial inflow but recurrent venous thrombosis. Mechanical thrombectomy provided only a transient improvement. On postoperative day 5, the platelet count fell to 42×10⁹/L with rapid clinical deterioration, including loss of intravenous access. A hematology consultation confirmed HIT with thrombosis; the 4Ts score was 8/8, supporting the immediate cessation of all heparin products and the initiation of non-heparin anticoagulation (Table 2).

**Table 2:** 4Ts score applied to this case Score range 0-8; 6-8: high pretest probability. Points assigned per the published 4Ts criteria [12]. In high probability, discontinue all heparin products and initiate a non-heparin anticoagulant while arranging confirmatory testing.

4Ts component	Observation in this case	Points (0-2)
Thrombocytopenia	Nadir 42×10⁹/L with >50% fall	2
Timing	Platelet fall by postoperative day 5 after perioperative heparin	2
Thrombosis	Recurrent venous thrombosis in flap veins	2
Other causes	No strong alternative cause identified	2
Total		8 (high)

The patient progressed to multiorgan failure and died on postoperative day 7.

## Discussion

HIT is a rare, immune-mediated adverse reaction to heparin that produces thrombocytopenia with a paradoxical prothrombotic state. In this case, thrombosis was present (i.e., HIT with thrombosis). This prothrombotic state is caused by IgG antibodies directed against PF4-heparin complexes, leading to platelet activation and widespread coagulation. Thrombosis occurs in up to 50% of patients with type II HIT and may involve both arterial and venous systems. Mortality can approach 30% in untreated cases [11,13]. In this patient, a high 4Ts score (Table 2; total 8/8) and the marked platelet fall (Table 1), together with early recurrent venous thrombosis despite preserved arterial inflow documented at re-exploration (Appendix D), are consistent with HIT as the proximate cause of free flap loss.

Two forms of HIT are recognized. Type I HIT is non-immune, transient, and generally harmless, occurring within the first 72 hours of heparin exposure. In contrast, type II HIT is an immune-mediated disorder that typically develops 4-10 days after heparin initiation. It is associated with life-threatening thromboembolic events and demands urgent intervention [14].

In our case, the clinical presentation was initially suggestive of mechanical venous compromise following fibular free flap reconstruction. As is often the case, systemic causes, such as HIT, were not considered until a profound drop in platelet count and progressive clinical deterioration prompted further investigation. The patient ultimately developed multiorgan failure and died. PF4-heparin antibody testing later returned positive. This case highlights a diagnostic blind spot in microsurgical reconstruction. Venous congestion is most often attributed to anastomotic issues, external compression, or technical error [15]. Systemic hypercoagulable conditions, particularly in otherwise healthy patients, may be overlooked. In such cases, routine assumptions can delay diagnosis and appropriate treatment.

The 4Ts scoring system provides a validated clinical tool to assess the likelihood of HIT, using the degree of thrombocytopenia, the timing of platelet drop, thrombosis, and other potential causes. A score of 6-8 is highly predictive [12]. Our patient scored 8/8, which, in hindsight, justified early intervention with alternative anticoagulation [16]. Laboratory confirmation of HIT involves PF4-heparin enzyme-linked immunosorbent assay (ELISA) testing (high sensitivity) and functional assays such as the serotonin release assay (high specificity). However, treatment must not be delayed pending results. Immediate cessation of all heparin products and initiation of non-heparin anticoagulants, such as argatroban or fondaparinux, are essential [17].

Postoperative thrombocytopenia has several differentials, including sepsis-associated disseminated intravascular coagulation, drug-induced thrombocytopenia, dilutional/consumptive etiologies, and marrow suppression [7,8,13]. In this patient, the >50% platelet fall to 42×10⁹/L after perioperative heparin, together with recurrent venous thrombosis despite preserved inflow, was most consistent with HIT (4Ts 8/8; Table 2) [12,13]. PF4-heparin ELISA was positive; however, the optical density and any functional assay results were unavailable, and daily platelet counts were not recorded. Accordingly, we present the available preoperative and postoperative day 5 counts (Table 1) and the procedural timeline (Appendix D). This interpretation is consistent with reports of HIT-associated free flap thrombosis in the microsurgery literature [5,18,19].

The limitations of this report are as follows: single-case design, lack of daily platelet trajectories, unavailable PF4 ELISA optical density and functional testing, absent precise timestamps for heparin dosing and for the initiation of non-heparin anticoagulation, and no intraoperative photodocumentation of retrieved thrombi. These gaps limit temporal certainty and the strength of causal inference.

Awareness of HIT in microsurgical reconstruction is critical. Unexplained or recurrent venous congestion should prompt routine platelet monitoring in the early postoperative period, early application of the 4Ts score, immediate cessation of all heparin (including flushes) when probability is intermediate to high, timely initiation of a non-heparin anticoagulant, and early hematology involvement.

## Conclusions

Pragmatically, persistent venous congestion with preserved inflow after perioperative heparin exposure should trigger a 4Ts assessment, immediate cessation of all heparin (including flushes), and initiation of a non-heparin anticoagulant while confirmatory testing is arranged and hematology is involved early.

## Figures and Tables

**Figure 3:**
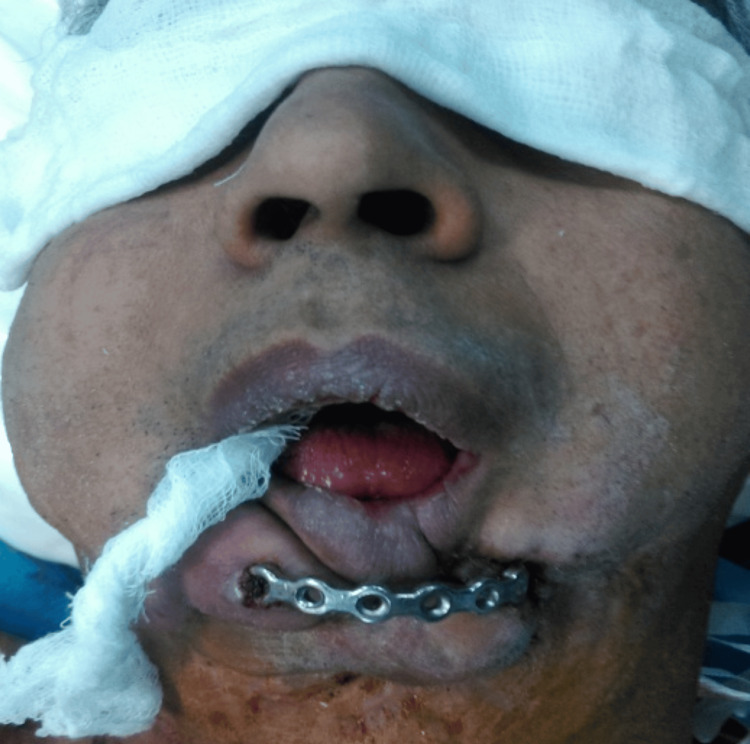
Preoperative extraoral view Frontal view showing the anterior mandibular defect and soft tissue loss prior to reconstruction.

**Figure 4:**
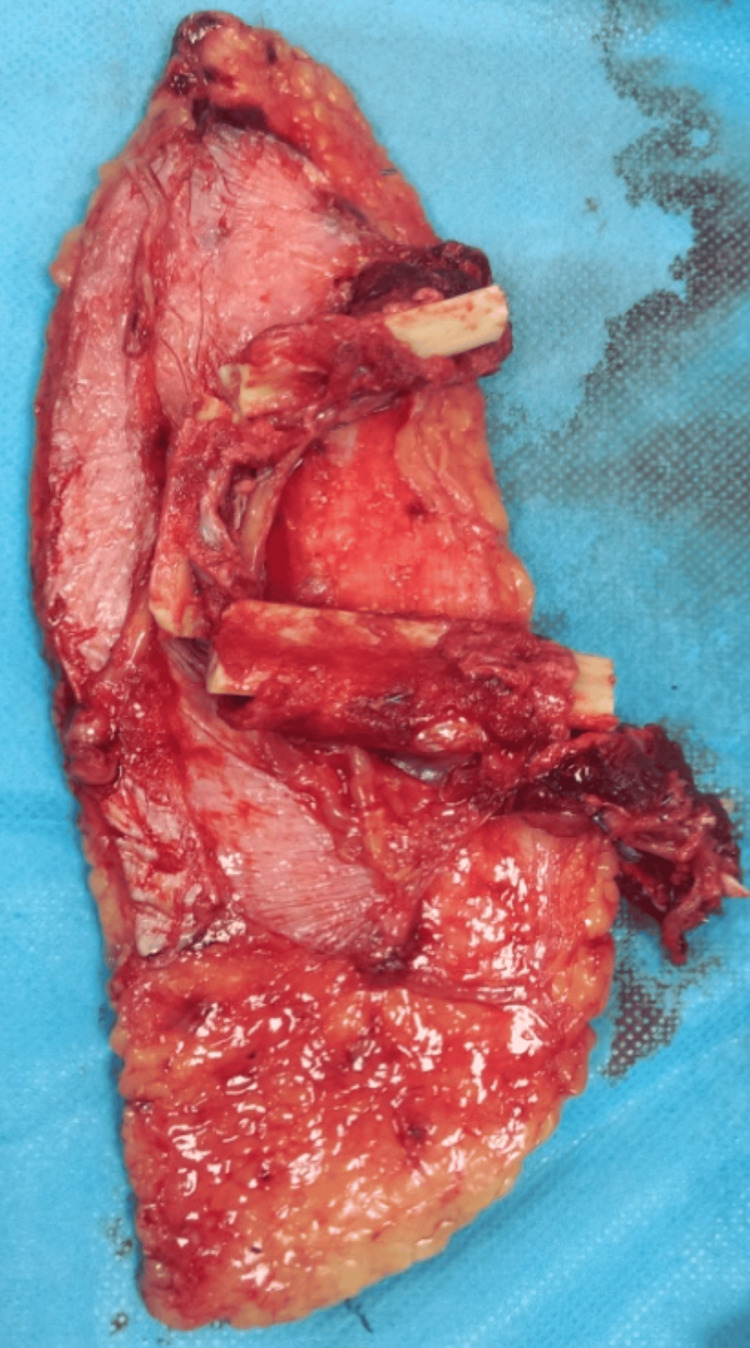
Back-table fibular osteocutaneous flap Skin paddle with osteotomized fibular segments prepared for mandibular inset; peroneal artery and venae comitantes preserved.

## Appendices

Appendix A

Appendix B

**Table 3:** Preoperative assessment summary ECG and chest radiograph were reviewed by anesthesia and surgical teams and documented as unremarkable in the perioperative record; images were not retained for publication. ECG: electrocardiogram; PA: posteroanterior; ASA: American Society of Anesthesiologists

Assessment	Finding	Notes
ECG (12-lead)	Normal sinus rhythm	Rate ~70-90 bpm; no ST-T changes
Chest radiograph (PA)	No acute cardiopulmonary process	Clear lungs; normal cardiac silhouette
Anesthesia evaluation	Cleared for surgery	ASA class II; no contraindications

Appendix C 

Appendix D

**Table 4:** Re-exploration log with operative findings Times are recorded in 24-hour format (local time). Anastomotic findings refer to the microvascular venous anastomosis (vena(e) comitans to the recipient vein). Actions included mechanical thrombectomy and anastomotic revision/irrigation as indicated. No intraoperative photodocumentation of retrieved thrombi was retained. POD: postoperative day; NS: normal saline; LR: lactated Ringer's

Date/time (POD)	Indication	Anastomotic findings	Action taken	Immediate outcome
POD 3: after morning round	Persistent venous congestion	Multiple intraluminal venous thrombi	Mechanical thrombectomy; anastomosis revision; NS/LR irrigation	Temporary improvement
POD 3: late afternoon	Recurrent congestion	Venous thrombosis	Thrombectomy; irrigation	Transient improvement
POD 4: pre-noon session	Recurrent congestion	Venous thrombosis	Thrombectomy	Limited improvement
POD 4: evening	Recurrent congestion	Venous thrombosis	Thrombectomy	No durable improvement
